# Supplementary figures and images for: Porcine placenta hydrolysate as an alternate functional food ingredient: In vitro antioxidant and antibacterial assessments
Source: PLoS One. 2021 Oct 25;16(10):e0258445. doi: 10.1371/journal.pone.0258445 (PMC8544860; doi:10.1371/journal.pone.0258445)

## Slide 1
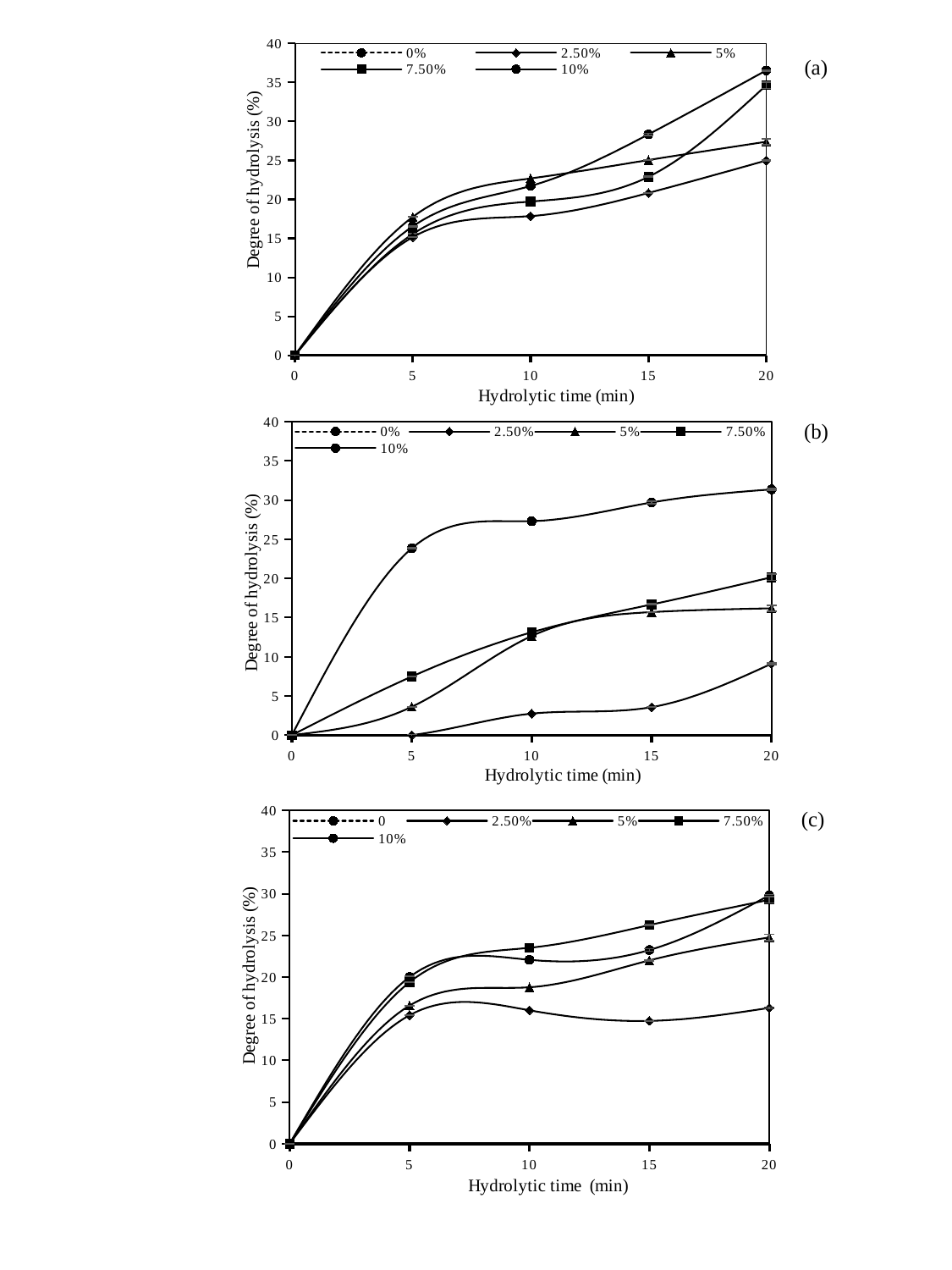

### Chart
| Category | 0% | 2.50% | 5% | 7.50% | 10% |
|---|---|---|---|---|---|(a)
### Chart
| Category | 0% | 2.50% | 5% | 7.50% | 10% |
|---|---|---|---|---|---|(b)
### Chart
| Category | 0 | 2.50% | 5% | 7.50% | 10% |
|---|---|---|---|---|---|(c)

Supplement: S1 Fig — (PPTX) [file pone.0258445.s001.pptx]

## Slide 1
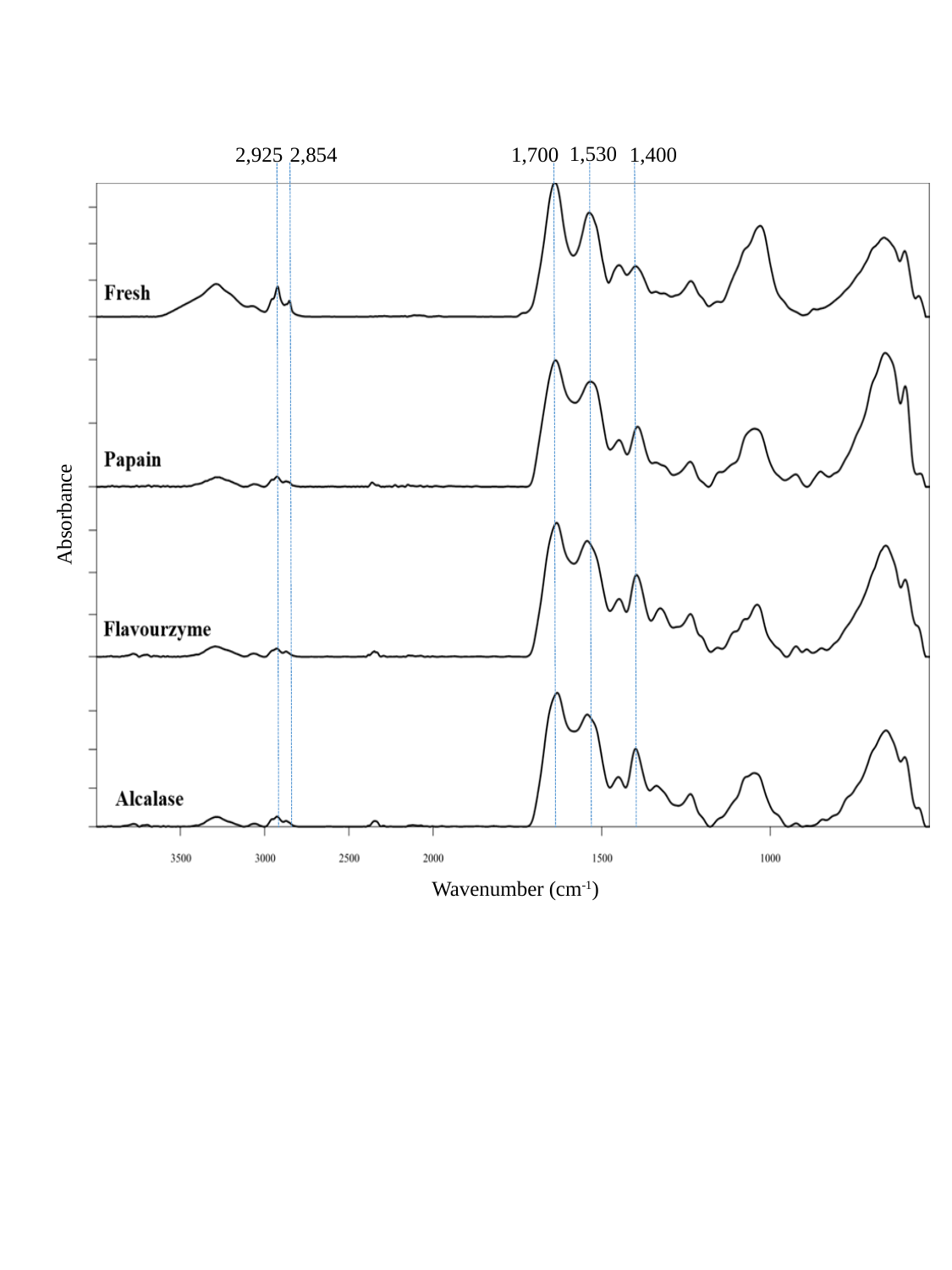

1,530
1,400
2,854
2,925
1,700
Absorbance
Wavenumber (cm-1)

Supplement: S6 Fig — (PPTX) [file pone.0258445.s006.pptx]
