# Supplementary material for: Porcine placenta hydrolysate as an alternate functional food ingredient: In vitro antioxidant and antibacterial assessments
Source: PLoS One. 2021 Oct 25;16(10):e0258445. doi: 10.1371/journal.pone.0258445 (PMC8544860; doi:10.1371/journal.pone.0258445)
Supplement: S2 Fig — (PPTX) [file pone.0258445.s002.pptx]

## Slide 1
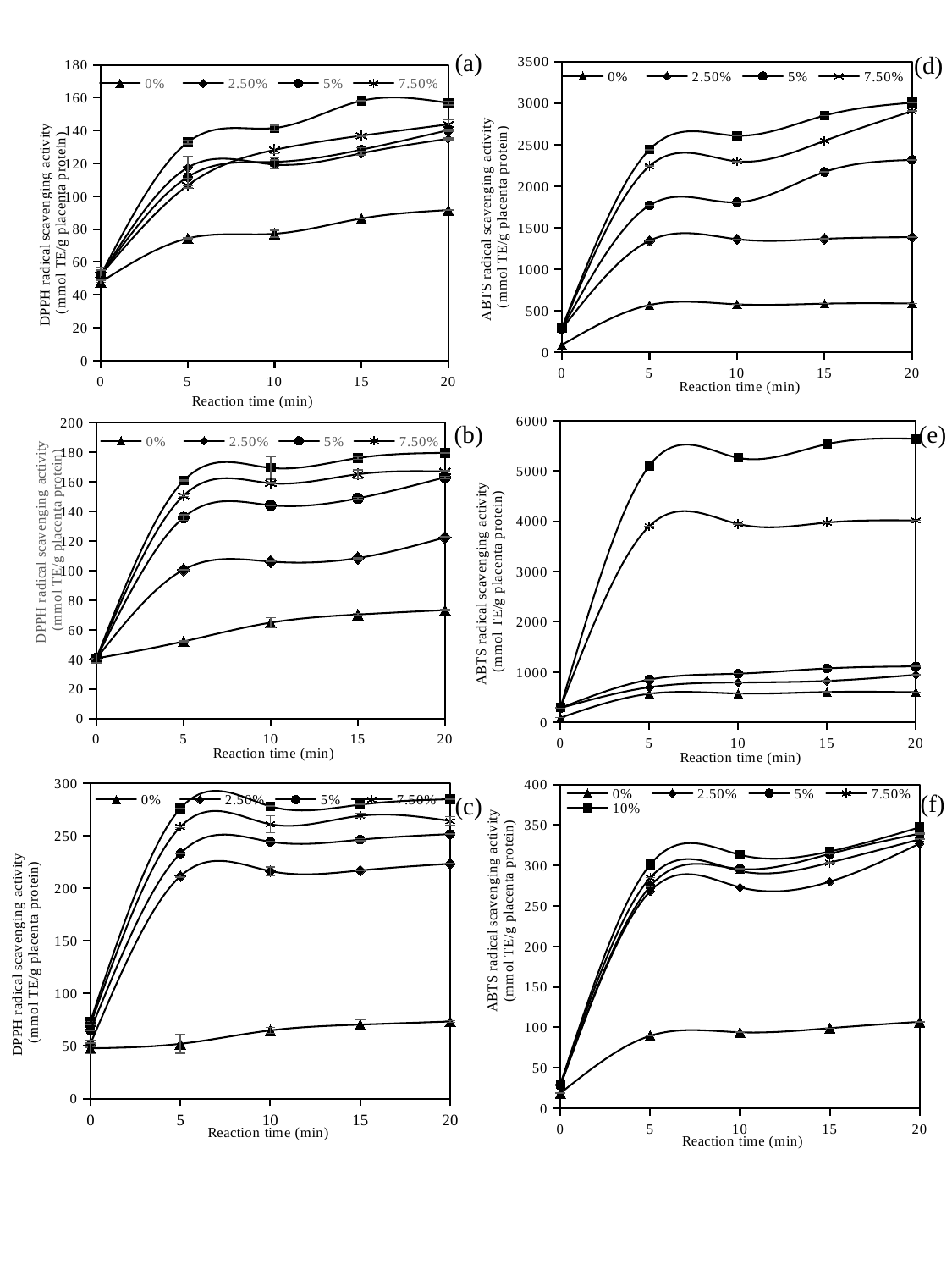

(a)
(d)
### Chart
| Category | 0% | 2.50% | 5% | 7.50% | 10% |
|---|---|---|---|---|---|
### Chart
| Category | 0% | 2.50% | 5% | 7.50% | 10% |
|---|---|---|---|---|---|
### Chart
| Category | 0% | 2.50% | 5% | 7.50% | 10% |
|---|---|---|---|---|---|(b)
(e)
### Chart
| Category | 0% | 2.50% | 5% | 7.50% | 10% |
|---|---|---|---|---|---|
### Chart
| Category | 0% | 2.50% | 5% | 7.50% | 10% |
|---|---|---|---|---|---|
### Chart
| Category | 0% | 2.50% | 5% | 7.50% | 10% |
|---|---|---|---|---|---|(f)
(c)
