# Supplementary material for: Porcine placenta hydrolysate as an alternate functional food ingredient: In vitro antioxidant and antibacterial assessments
Source: PLoS One. 2021 Oct 25;16(10):e0258445. doi: 10.1371/journal.pone.0258445 (PMC8544860; doi:10.1371/journal.pone.0258445)
Supplement: S4 Fig — (PPTX) [file pone.0258445.s004.pptx]

## Slide 1
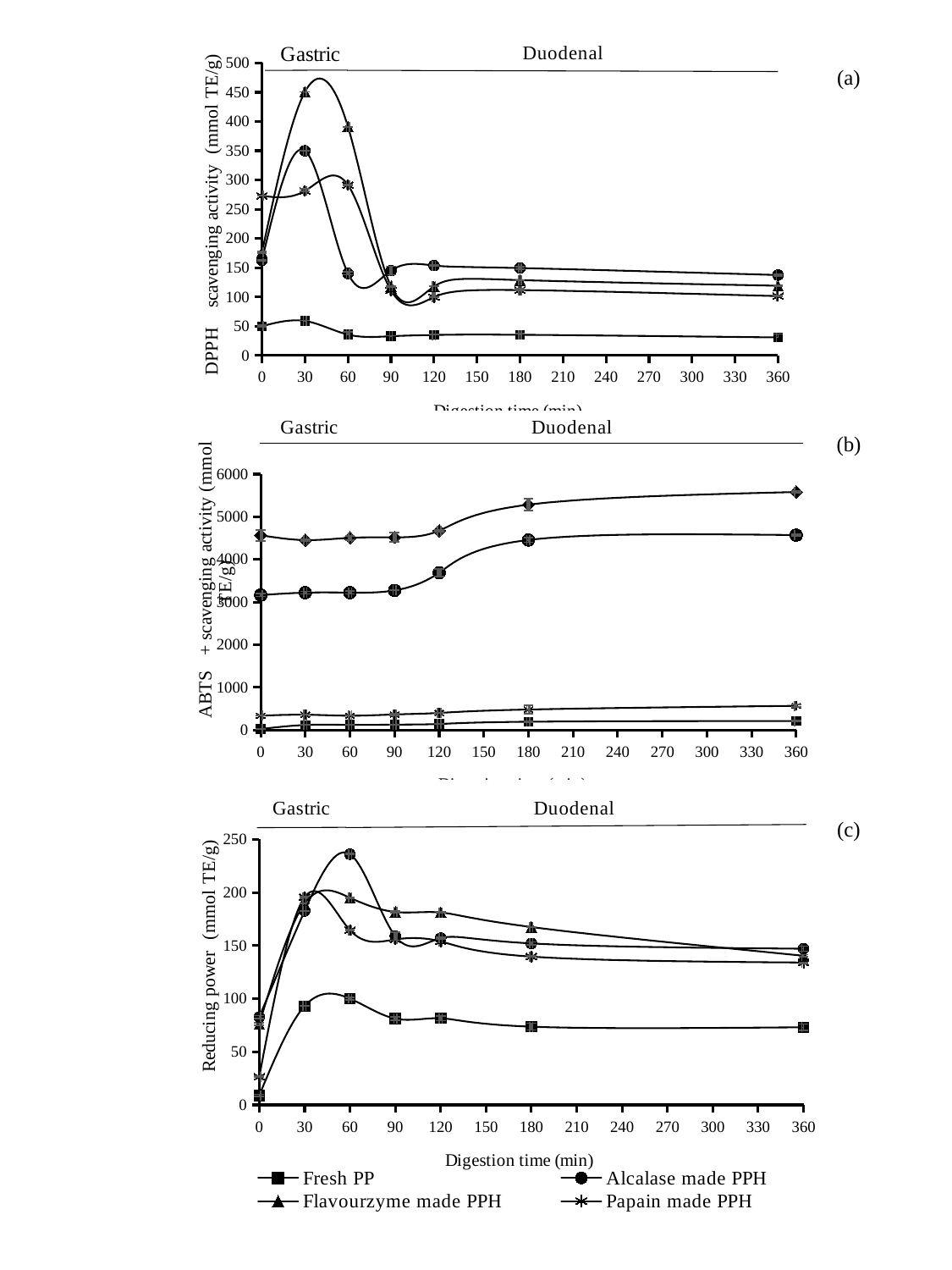

### Chart
| Category | Flavourzyme made PPH | Alcalase made PPH | Papain made PPH | Fresh PP |
|---|---|---|---|---|(a)
### Chart
| Category | Fresh PP | Alcalase made PPH | Flavourzyme made PPH | Papain made PPH |
|---|---|---|---|---|(b)
### Chart
| Category | Fresh PP | Alcalase made PPH | Flavourzyme made PPH | Papain made PPH |
|---|---|---|---|---|(c)
