# Supplementary material for: Porcine placenta hydrolysate as an alternate functional food ingredient: In vitro antioxidant and antibacterial assessments
Source: PLoS One. 2021 Oct 25;16(10):e0258445. doi: 10.1371/journal.pone.0258445 (PMC8544860; doi:10.1371/journal.pone.0258445)
Supplement: S5 Fig — (PPTX) [file pone.0258445.s005.pptx]

## Slide 1
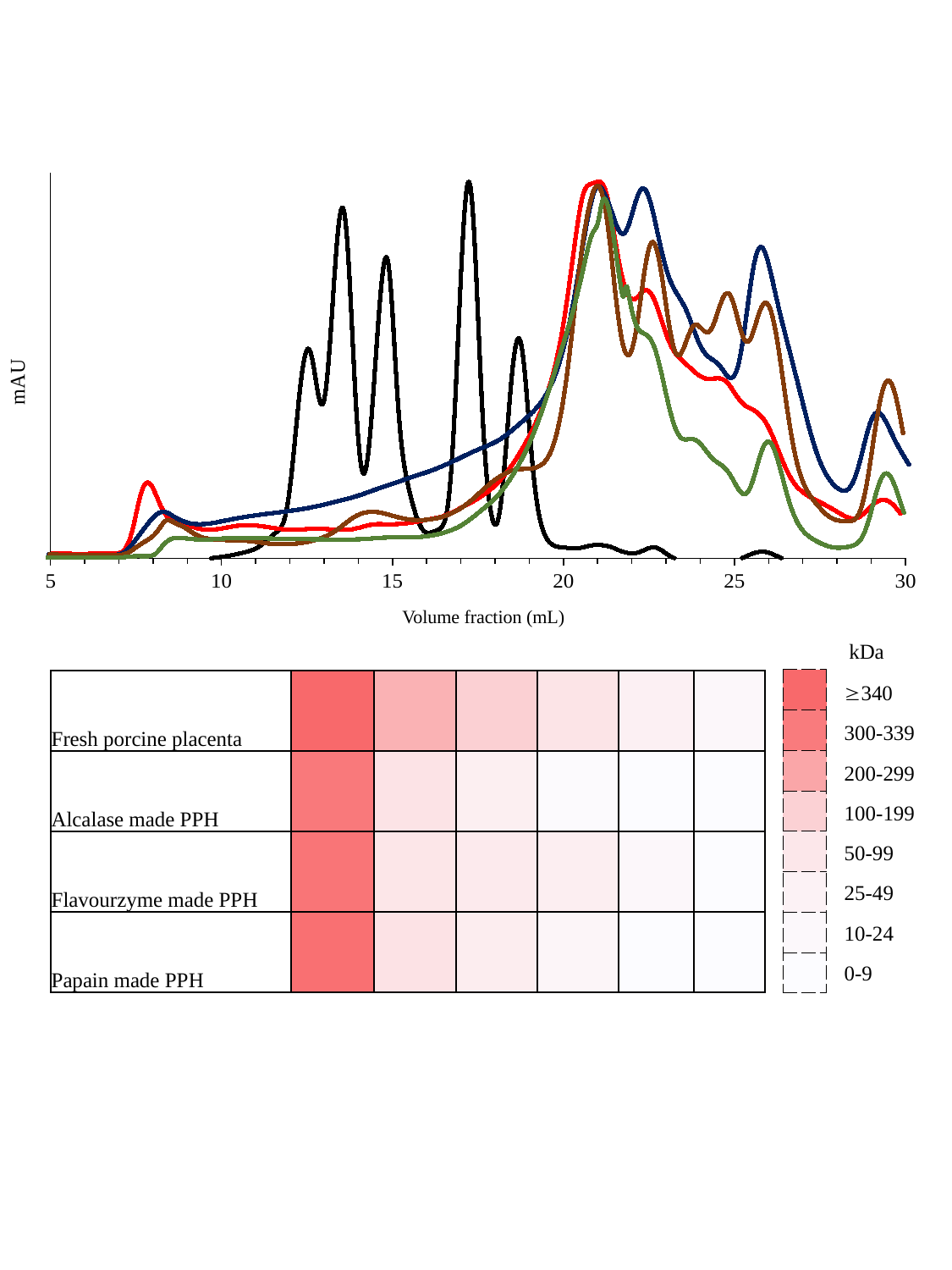

### Chart
| Category | Fresh PP |
|---|---|
### Chart
| Category | Flavourzyme made PPH |
|---|---|
### Chart
| Category | Alcalase made PPH |
|---|---|
### Chart
| Category | Papain made PPH |
|---|---|
### Chart
| Category | mAU |
|---|---|
Volume fraction (mL)
kDa
| |
| --- |
| |
| |
| |
| |
| |
| |
| |
| Fresh porcine placenta | | | | | | |
| --- | --- | --- | --- | --- | --- | --- |
| Alcalase made PPH | | | | | | |
| Flavourzyme made PPH | | | | | | |
| Papain made PPH | | | | | | |
| ≥340 |
| --- |
| 300-339 |
| 200-299 |
| 100-199 |
| 50-99 |
| 25-49 |
| 10-24 |
| 0-9 |
