# Supplementary material for: Porcine placenta hydrolysate as an alternate functional food ingredient: In vitro antioxidant and antibacterial assessments
Source: PLoS One. 2021 Oct 25;16(10):e0258445. doi: 10.1371/journal.pone.0258445 (PMC8544860; doi:10.1371/journal.pone.0258445)
Supplement: S1 Table — (DOCX) [file pone.0258445.s007.docx]

**Table 1** Proximate composition, TCA-soluble peptide, mineral profile, and amino acid composition of freeze-dried porcine placenta

| Composition | Contents |
| --- | --- |
| Proximate composition (%) |  |
| Moisture | 6.61±0.03 |
| Protein | 78.03±0.30 |
| Fat | 2.40±0.40 |
| Crude fiber | 0.61±0.01 |
| Ash | 8.59±0.99 |
| Carbohydrate | 3.34±0.16 |
| TCA-soluble peptide (mmol tyrosine/g) | 48.12±0.77 |
| Minerals (mg/100 g) |  |
| Calcium | 1789.09±51.58 |
| Phosphorus | 1052.54±29.01 |
| Sodium | 571.49±11.89 |
| Potassium | 224.42±0.79 |
| Magnesium | 61.64±1.23 |
| Iron | 20.76±0.20 |
| Zinc | 5.54±0.10 |
| Manganese | 0.81±0.03 |
| Copper | 0.84±0.01 |
| Amino acid composition (g/100 g) |  |
| Essential animo acid (EAA) |  |
| Leucine | 5.73±0.23 |
| Lysine | 4.05±0.33 |
| Valine | 3.98±0.25 |
| Phenylalanine | 2.76±0.08 |
| Threonine | 2.70±0.06 |
| Histidine | 1.82±0.20 |
| Isoleucine | 1.78±0.06 |
| Methionine | 1.07±0.04 |
| Tryptophan | 0.71±0.01 |
| Non-essential amino acid (NEAA) |  |
| Glutamic acid | 9.73±0.33 |
| Proline | 8.70±0.17 |
| Glycine | 8.49±0.29 |
| Aspartic acid | 6.85±0.12 |
| Alanine | 4.80±0.19 |
| Arginine | 4.48±0.15 |
| Hydroxyproline | 3.89±0.46 |
| Serine | 3.67±0.15 |
| Tyrosine | 2.09±0.17 |
| Cysteine | 0.91±0.02 |
| Cystine | 0.62±0.01 |

Values are given as mean ± standard deviation from triplicate determinations.
