# Supplementary material for: Porcine placenta hydrolysate as an alternate functional food ingredient: In vitro antioxidant and antibacterial assessments
Source: PLoS One. 2021 Oct 25;16(10):e0258445. doi: 10.1371/journal.pone.0258445 (PMC8544860; doi:10.1371/journal.pone.0258445)
Supplement: S2 Table — (DOCX) [file pone.0258445.s008.docx]

**Table 2** Correlation coefficient of enzymatic hydrolytic variables and antioxidant activities of porcine placenta hydrolysate.

| Enzyme | Variable | DPPH^•^ scavenging activity | ABTS^•+^ scavenging activity | Metal chelating capacity | Reducing power |
| --- | --- | --- | --- | --- | --- |
| Alcalase | Hydrolysis time | 0.4035 | 0.2059 | 0.5050 | 0.3817 |
|  | Enzyme load | 0.7517 | 0.9462 | 0.7512 | 0.7583 |
|  | DH | 0.9400 | 0.8308 | 0.9496 | 0.9362 |
| Flavourzyme | Hydrolysis time | 0.3480 | 0.0619 | 0.4417 | 0.2059 |
|  | Enzyme load | 0.8757 | 0.9166 | 0.7522 | 0.8647 |
|  | DH | 0.8482 | 0.7181 | 0.9301 | 0.7038 |
| Papain | Hydrolysis time | 0.0651 | 0.1514 | 0.3044 | 0.1622 |
|  | Enzyme load | 0.9084 | 0.7436 | 0.8854 | 0.9515 |
|  | DH | 0.9563 | 0.9205 | 0.9650 | 0.8830 |

DH = degree of hydrolysis
