# Supplementary material for: Porcine placenta hydrolysate as an alternate functional food ingredient: In vitro antioxidant and antibacterial assessments
Source: PLoS One. 2021 Oct 25;16(10):e0258445. doi: 10.1371/journal.pone.0258445 (PMC8544860; doi:10.1371/journal.pone.0258445)
Supplement: S3 Table — (DOCX) [file pone.0258445.s009.docx]

**Table 3** Antibacterial activity (zone of inhibition) of porcine placenta hydrolysate (PPH) catalysed by three different enzymes at 10% enzyme concentration for 20 min.

| Sample | Clear zone (mm) | |
| --- | --- | --- |
|  | *S.aureus* | *E.coli* |
| Fresh placenta | 2.30±0.78^a^ | 1.80±0.00^a^ |
| Alcalase made PPH | 3.52±0.19^b^ | 5.00±0.38^b^ |
| Flavourzyme made PPH | 2.50±0.45^a^ | 2.80±0.94^a^ |
| Papain made PPH | 10.70±0.17^c^ | 7.80±0.74^c^ |

Values are given as mean ± standard deviation from six determinations.

Different letters in the same row indicate significant differences (*p* < 0.05).
