# Supplementary material for: Porcine placenta hydrolysate as an alternate functional food ingredient: In vitro antioxidant and antibacterial assessments
Source: PLoS One. 2021 Oct 25;16(10):e0258445. doi: 10.1371/journal.pone.0258445 (PMC8544860; doi:10.1371/journal.pone.0258445)
Supplement: Attachment — Submitted filename: Response to Reviewers.docx [file pone.0258445.s011.docx]

**Response to Reviewers**

All points raised by the reviewers were carefully addressed and answered point-by-point. A revision was made in highlighted red fonts. The revised manuscript was carefully prepared to meet PLOS ONE's style requirements.

**Journal Requirements:**

When submitting your revision, we need you to address these additional requirements.

1. Please ensure that your manuscript meets PLOS ONE's style requirements, including those for file naming. The PLOS ONE style templates can be found at

https://journals.plos.org/plosone/s/file?id=wjVg/PLOSOne_formatting_sample_main_body.pdf and

https://journals.plos.org/plosone/s/file?id=ba62/PLOSOne_formatting_sample_title_authors_affiliations.pdf

Ans: The revised manuscript was carefully prepared to meet PLOS ONE's style requirements.

2. PLOS requires an ORCID iD for the corresponding author in Editorial Manager on papers submitted after December 6th, 2016. Please ensure that you have an ORCID iD and that it is validated in Editorial Manager. To do this, go to ‘Update my Information’ (in the upper left-hand corner of the main menu), and click on the Fetch/Validate link next to the ORCID field. This will take you to the ORCID site and allow you to create a new iD or authenticate a pre-existing iD in Editorial Manager. Please see the following video for instructions on linking an ORCID iD to your Editorial Manager account: https://www.youtube.com/watch?v=_xcclfuvtxQ

Ans: The ORCID iD for the corresponding author is given.

3. We suggest you thoroughly copyedit your manuscript for language usage, spelling, and grammar. If you do not know anyone who can help you do this, you may wish to consider employing a professional scientific editing service.

Ans: QuillBot, a paraphrase tool, was used to double-check the language throughout the manuscript.

**Reviewer #1:** The presented manuscript, "Porcine placenta hydrolysate as an alternate functional food ingredient" seems okay. It will be very good if the authors go for thorough language checking prior publishing the article.

Ans: QuillBot, a paraphrase tool, was used to double-check the language.

**Reviewer #2:** The manuscript entitled "Porcine placenta hydrolysate as an alternate functional food ingredient" describes the food functional values and antioxidant activities of Porcine placenta hydrolysate. The manuscript has been nicely represented with significant scientific work. Some figures are not clear to understand properly. English editing is needed since there are many Grammatical errors in the manuscript. Statistical significance should be included in the figure 1 to figure 4. After these changes, manuscript may be accepted for the publication.

Ans: The Preflight Analysis and Conversion Engine (PACE) digital diagnostic tool, https://pacev2.apexcovantage.com/, was used to check the quality of all figures. We double-checked that the figures complied with PLOS guidelines. QuillBot, a paraphrase tool, was used to double-check the language. All figures and tables were statistically analyzed, as stated in the text. The letters indicating statistical differences, on the other hand, were not included in the figures since they would make the figures too cluttered.

Reviewer #3: The work done by Worawan Panpipat et al., entitled “Porcine placenta hydrolysate as an alternate functional food ingredient” is too preliminary for any scientific conclusion. There are few comments that need to be properly addressed before further progress of the manuscript.

1.The author should find out the effect of hydrolysate in in vivo/ in vitro model.

Ans: The statement and its significance were given in the Abstract. “*The production of bioactive peptides from animal-based raw materials highly depends on enzymatic hydrolysis. Porcine placenta is an underutilized biomass in Thailand's pig farms, yet it is still a source of proteins and beneficial compounds. Porcine placenta could be used as a protein substrate for the production of enzymatic hydrolysate, which could be employed as a functional food ingredient in the future. The goal of this study was to enzymatically produce porcine placenta hydrolysates (PPH) using three commercial enzymes (Alcalase, Flavouzyme, and papain) and evaluate their in vitro antioxidant and antibacterial activity.*”. As a result, the antioxidant and antibacterial properties were investigated *in vitro* in this work. This study did not contain any *in vivo* testing. So the title was changed to “*Porcine placenta hydrolysate as an alternate functional food ingredient: in vitro antioxidant and antibacterial assessments*” to make it more apparent. *In vitro* tests can be performed to examine the bioactivity of active compounds such as protein hydrolysate, which is particularly useful in the area of Food Science and Technology.

2. The author should also perform some additional experiment to established anti-microbial and antioxidative properties.

Ans: The antibactial and antioxidant capabilities were tested and reported in the original manuscript. The antioxidant activities were exhibited, which included DPPH^•^/ABTS^•+^ scavenging activities (Fig. 2), reducing power (Fig. 3) and metal chelating activity (Fig. 3). Table 2 also includes the correlation coefficient between enzymatic hydrolytic variables and antioxidant activities of porcine placenta hydrolysate. In addition, the gastrointestinal (GI) tract model system was used to investigate changes in DPPH^•^/ABTS^•+^ scavenging activities and reducing power. The antibacterial activity of PPH was examined using the agar-well diffusion method against *Staphylococcus aureus* and *Escherichia coli*, and the zone of inhibition was reported. All of the tests can be used to draw conclusions about porcine placenta hydrolysate's antioxidant and antibacterial activities.

3.The author should provide appropriate figure legends.

Ans. All of the legends for the figures were double-checked.

4.Author should go through the manuscript thoroughly to check the typographical errors and

grammatical mistakes.

Ans: QuillBot, a paraphrase tool, was used to check for typographical errors and recheck the language.

Reviewer #4:

1. Restructure Ln 52, 53, 54

Ans: It was changed to “*Wastes and by-products are being reassembled in the farrow-to-finish production pig farming system, which consists of breeding pigs, producing piglets, and fattening pigs, as a result of the expansion of large-scale pig farms [3].*”

2. Ln 56, 57 do not have citation

Ans: Because no research has been documented in Thailand, no references were provided. Therefore, the sentence was changed to “*However, there has been no published research on the use of pig placenta in Thailand. It is of great interest to figure out how to use the right technologies to add value to this placenta.*”

3. Ln 63, 63 has poor English, need correction

Ans: It was changed to “*Collagen is the most abundant protein in the extracellular matrix of the placenta, with lately high market values [4, 5, 6].*”

4. rationale of the study is not appropriate in the given interest

Ans: The rationale of the study was intensively revised as highlighted in the Introduction.

5. Mention Cat# for all the reagents/Chemicals, and other consumables.

Ans: It was stated in the “Chemical” section that “*All enzymes (Alcalase from Bacillus licheniformis, Flavourzyme from Aspergillus oryzae protease, papain from papaya latex) and chemicals (e.g., trichloroacetic acid (TCA), 2,4,6-trinitrobenzene sulfonic acid (TNBS), 2,2-diphenyl-1-picrylhydrazyl (DPPH), 3-(2-pyridyl)-5-6-diphenyl-1,2,4-triazine-4′,4′′-disulphonic acid sodium salt (ferrozine), 2,2-azino-bis(3-thylbenzothiazoline-6-sulphonicacid) diammonium salt (ABTS), 2,4,6-tripyridyl-s-triazine (TPTZ)) were purchased from Sigma-Aldrich (St. Louis, MO, USA).*”. It met the journal's requirements, and many standard journals do not require the Cat#. As a result, we didn't include the Cat#. Thank you very much.

6. All experiments were carried out in triplicate (N = 3), does it satisfy power of the study

Ans: All experiments were run in triplicate with three different lots of placenta (N = 3). As a result, the study's power was satisfied.

7. Figure Legends needs to re-write and not conveying required information

Ans: The figure legends were revised accordingly.

8. anti-microbial and anti-oxidant properties are not established properly

Ans: The antioxidant activities were tested, which included DPPH^•^/ABTS^•+^ scavenging activities (Fig. 2), reducing power (Fig. 3) and metal chelating activity (Fig. 3). Table 2 also includes the correlation coefficient between enzymatic hydrolytic variables and antioxidant activities of porcine placenta hydrolysate. In addition, the gastrointestinal (GI) tract model system was used to investigate changes in DPPH^•^/ABTS^•+^ scavenging activities and reducing power. The antibacterial activity of PPH was examined using the agar-well diffusion method against *Staphylococcus aureus* and *Escherichia coli*, and the zone of inhibition was reported. All of the tests can be used to draw conclusions about porcine placenta hydrolysate's antioxidant and antibacterial activities.

While revising your submission, please upload your figure files to the Preflight Analysis and Conversion Engine (PACE) digital diagnostic tool, https://pacev2.apexcovantage.com/. PACE helps ensure that figures meet PLOS requirements. To use PACE, you must first register as a user. Registration is free. Then, login and navigate to the UPLOAD tab, where you will find detailed instructions on how to use the tool. If you encounter any issues or have any questions when using PACE, please email PLOS at figures@plos.org. Please note that Supporting Information files do not need this step.

Ans: Done.
